# A Novel and Divergent Gyrovirus with Unusual Genomic Features Detected in Wild Passerine Birds from a Remote Rainforest in French Guiana

**DOI:** 10.3390/v11121148

**Published:** 2019-12-11

**Authors:** Daniel A. Truchado, José Manuel Diaz-Piqueras, Esperanza Gomez-Lucia, Ana Doménech, Borja Milá, Javier Pérez-Tris, Jonas Schmidt-Chanasit, Daniel Cadar, Laura Benítez

**Affiliations:** 1 Department of Genetics, Physiology and Microbiology, Complutense University of Madrid, Calle José Antonio Novais 12, 28040 Madrid, Spain; danieltr@ucm.es (D.A.T.); josemand@ucm.es (J.M.D.-P.); lbenitez@ucm.es (L.B.); 2 Department of Biodiversity, Ecology and Evolution Faculty of Biology, Complutense University of Madrid, Calle José Antonio Novais 12, 28040 Madrid, Spain; jperez@ucm.es; 3 Department of Animal Health, Faculty of Veterinary Medicine, Complutense University of Madrid, Avda. Puerta de Hierro s/n, 28040 Madrid, Spain; duato@ucm.es (E.G.-L.); domenech@vet.ucm.es (A.D.); 4 National Museum of Natural Sciences, Spanish National Research Council (CSIC), Calle José Gutiérrez Abascal 2, 28006 Madrid, Spain; borjamila@gmail.com; 5 Bernhard Nocht Institut for Tropical Medicine, WHO Collaborating Centre for Arbovirus and Haemorrhagic Fever Reference and Research, National Reference Centre for Tropical Infectious Diseases, Bernhard-Nocht-Strasse 74, 20359 Hamburg, Germany; jonassi@gmx.de; 6 University of Hamburg, Faculty of Mathematics, Informatics and Natural Sciences, Mittelweg 177, 20148 Hamburg, Germany

**Keywords:** gyrovirus, passerines, French Guiana, metagenomics, tropical rainforest

## Abstract

Sequence-independent amplification techniques have become important tools for virus discovery, metagenomics, and exploration of viral diversity at the global scale, especially in remote areas. Here, we describe the detection and genetic characterization of a novel gyrovirus, named GyV11, present in cloacal, oral, and blood samples from neotropical wild birds in French Guiana. The molecular epidemiology revealed the presence of GyV11 only in passerine birds from three different species at a low prevalence (0.73%). This is the first characterization and prevalence study of a gyrovirus carried out in resident wild bird populations in a remote region, and provides evidence of the fecal–oral route transmission and local circulation of the virus. The molecular phylogeny of gyroviruses reveals the existence of two distinct gyrovirus lineages in which GyV11 is phylogenetically distinct from previously reported gyroviruses. Furthermore, GyV11 is placed basal in the gyrovirus phylogeny, likely owing to its ancestral origin and marked divergence. This study also provides important insights into the ecology, epidemiology, and genomic features of gyroviruses in a remote neotropical rainforest. The pathogenesis of this virus in avian species or whether GyV11 can infect humans and/or chickens needs to be further investigated.

## 1. Introduction

In recent years, numerous novel viruses and virus variants have been discovered by virus metatranscriptomics and metagenomics in different bird species, mainly in wild birds [1,2,3]. Nevertheless, there is still very little known about viruses that circulate in wild birds, especially in remote areas, and their possible interaction with domestic fowl or even their zoonotic potential. Next generation sequencing (NGS) technology allows the identification of, in principle, all the viruses present in a given sample, which improves our knowledge on the viral diversity and evolution in that area and helps estimate the potential risk to domestic fowl or humans [4].

Gyroviruses (GyVs) are non-enveloped icosahedral viruses whose genome is a circular, single-stranded DNA molecule of approximately 2 Kb. They are grouped in the genus *Gyrovirus*, which has been recently reclassified in the *Anelloviridae* family [5]. Chicken anemia virus (CAV) is so far known to be the only pathogenic member of the genus and has long been the prototype and sole member of the genus *Gyrovirus* [5]. CAV was first reported in 1979 affecting domestic chickens (*Gallus gallus domesticus*) [6], and its infection is an economically important clinical and subclinical disease in young chickens, with a worldwide distribution [7]. The other members of the genus *Gyrovirus* are GyV2 through GyV10 and the human gyrovirus (HGyV1). HGyV1 was identified in healthy French blood donors, as well as in blood samples from solid organ transplant recipients and in an HIV-infected person from Italy [8,9]. This genus includes very divergent members, mainly isolated from chicken faeces, meat, or brain tissue [10,11,12], as well as from human skin, faeces, and blood [13,14,15] and faeces of cats [12] and ferrets [16]. CAV, GyV2, and GyV3 species have also been detected in fecal samples from wild birds in Brazil [3]. Therefore, only three gyroviruses have been found exclusively in bird species different from chicken: GyV8, GyV10, and ASPaGyV. GyV8 was isolated from the spleen and uropygial gland of a northern fulmar (*Fulmarus glacialis*) with neurological clinical signs [17]. GyV10 was present in several crested screamers (*Chauna torquata*), some with neurologic disease and clinical and pathological features resembling CAV infection, as well as from apparently healthy birds [18]. The latest gyrovirus discovered, ASPaGyV, was detected in a metagenomic analysis from cloacal samples in ashy strom petrels (*Oceanodroma homochroa*) [19].

The pathogenic significance of these genetically novel avian and human gyroviruses is currently uncertain [5,20], but similarities between avian and human sequences suggest that some of them may have zoonotic potential [20]. This is highlighted by the observation of possible recombinants between strains from different origins [12]. In this study, we describe using unbiased deep sequencing the detection and genetic characterization of a novel gyrovirus species, designated as GyV11, from neotropical birds in French Guiana.

## 2. Materials and Methods

### 2.1. Sample Collection

The study was carried out at the Pararé and Inselberg field stations in the Nouragues Ecological Research Station, managed by France’s Centre National de la Recherche Scientifique (CNRS) and located in the Nouragues Natural Reserve in French Guiana. Birds of the understory were mist-netted in January 2016 (rainy season) and in October–November 2016 (dry season). From each bird, a small amount of blood, which was subsequently used for several preparations, was collected. After making a blood smear, the remaining volume was divided into two different tubes, one containing ethanol and the other RNAlater (Ambion, Life Technologies, Carlsbad, CA, USA). In addition, oral and cloacal swabs were taken from each individual. Cloacal swabs were not taken when the size of the bird was too small, mainly in hummingbirds. Both oral and cloacal samples were placed in 800 µL of universal viral transport medium (VTM) (Becton Dickinson). Blood samples in ethanol and RNAlater were stored at −20 °C, while oral and cloacal swabs were kept at −80 °C. A total of 406 birds from 72 species and 24 different families were sampled.

### 2.2. Sample Processing and Next Generation Sequencing

Cloacal samples with abundant fecal matter from 50 individual birds, including the highest bird species diversity possible (31 species of 12 families), were grouped in five pools of 10 individuals. The samples were subjected and processed for next-generation sequencing, as described elsewhere [21]. Shortly after, the faecal samples were vortexed and the swabs squeezed to release epithelial cells. The pellets obtained were resuspended in 250 µL PBS and subjected to two freeze–thaw cycles at −80 °C to maximize the release of viral particles. Afterwards, in order to reduce the volume of bacteria and other contaminants, the samples were filtered through 0.45 µm pore-sized columns. Filtrate of each sample (50 µL) was mixed in five pools of 10 samples each. Each filtrate was treated with a mixture of nucleases (Turbo DNase, Ambion, Carlsbad, CA, USA; Baseline-ZERO, Epicenter, Madison, WI, USA; Benzonase, Novagen, San Diego, CA, USA; RNAse One, Promega, Fitchburg, WI, USA) to digest unprotected nucleic acids including host DNA/RNA. Finally, viral RNA/DNA was extracted with the MagMAX Viral RNA Isolation Kit (Thermo Fisher) according to the manufacturer’s instructions. After random RT-PCR amplification, the extracted viral nucleic acids were subjected to library preparation using a QIAseq FX DNA Library Kit (Qiagen, Hilden, Germany) and sequenced using (2 × 300 bp paired-end) MiSeq Reagent Kits v3 (Illumina, San Diego, CA, USA) on a MiSeq platform. Raw reads were first trimmed and filtered to remove polyclonal and low-quality reads (<50 bases long) using CLC workbench (Qiagen). The remaining filtered raw reads were de novo assembled separately using Trinity v2.6. [22] and CLC workbench and compared with a non-redundant and viral proteome database using BLASTx with a cut-off E-value of 0.001. The virus-like contigs and singlets were further compared to all protein sequences in non-redundant protein databases with a default E-value cutoff of 0.001. The viral metagenomic and metatranscriptomic output was visualized and analyzed in MEGAN [23].

### 2.3. Prevalence of the Novel GyV11 in Cloacal, Oral, and Blood Samples

We tested for the presence of GyV11 in 356 additional cloacal samples that were not subjected to deep sequencing, and also in oral and blood samples from the 50 individuals whose cloacal samples were subjected to deep sequencing. Oral and blood samples of those individuals that tested positive in the cloacal screening were also analyzed. Cloacal samples were analyzed in pools, while oral and blood samples were analyzed individually.

The 50 selected individuals pooled for deep sequencing were screened by PCR for the presence of different viruses in their cloacal, oral, and blood samples. In order to detect the presence of GyV11 in our samples, we designed two sets of specific primer pairs: Gyr1168F (5′–GCATCCTGGCTTCACTCCTCACA–3′)/Gyr1168R (5′–CGGCGTCCCCTGCTGCTA–3′) and Gyr266F (5′–CCGCCGCGCTGGGAGGTA–3′)/Gyr266R (5′–TTGGCGTCTGAAGCGTTGAT–3′), which amplify genome fragments of 396 bp and 361 bp, respectively. In total, 406 birds were tested for the presence of the novel gyrovirus (Table S1).

All DNA amplifications were performed in 25 µL of reaction mixture, containing 0.625 U of AmpliTaq DNA polymerase (Applied Biosystems, Foster City, CA), PCR Buffer II (Applied Biosystems) supplemented to a final 2.5 mM MgCl_2_, 0.8 mM of each desoxynucleotide diphosphate (Fermentas, Vilnius, Lithuania), 10 pmol/µL of each primer, and 5 μL of extracted RNA/DNA. In the case of cloacal samples, we added 5 μL of 10^−1^ dilution from the RNA/DNA extraction owing to the presence of PCR inhibitors in faeces. The reactions comprised a first denaturation step at 94 °C for 5 min, 45 cycles (denaturation at 94 °C for 30 s, annealing at 57 °C for 30 s for both primer pairs, and polymerization at 72 °C for 30 s), and a final extension step at 72 °C for 5 min. RT-PCR was carried out using Verso 1-Step RT-PCR Kit (Thermo Fisher) following the manufacturer’s protocol.

### 2.4. Genomic Characterization and Phylogeny

The putative ORFs in the genome of the novel gyrovirus were predicted using ORFfinder and later identified by BLASTX. Pairwise similarities for the complete genome of the gyrovirus, putative ORFs, and protein sequences were calculated with Clustal Omega. To identify the main protein motifs, we applied Motif Scan (MyHits, SIB, Switzerland), cNLS Mapper [24], and NetNES 1.1 Server [25]. Heatmaps for protein pairwise similarities were created with Morpheus (https://software.broadinstitute.org/morpheus). Putative binding sites of the non-translated region (NTR) were identified using TFBIND [26] and GPMiner (http://gpminer.mbc.nctu.edu.tw), and stem-loop structures were analyzed using Mfold [27]. Tandem repeats were searched using Tandem repeats Finder [28]. We plotted the genome organization of the novel gyrovirus employing the DNAPlotter software [29].

The genome obtained for GyV11 was compared with all complete gyrovirus VP1, VP2, and VP3 protein sequences publicly available. Sequences were aligned using the MAFFT algorithm and then visually inspected in Geneious v9.1.4. To investigate the phylogenetic relationship of the GyV11 with other gyroviruses available in databases, phylogenetic reconstructions were performed using Bayesian inference and a Monte Carlo Markov chain (MCMC) sampling method as implemented in BEAST v.1.8.3 [30], and in parallel using maximum likelihood inference in PhyML v3.1 [31] based on VP1, VP2, and VP3 protein sequences. Analyses were performed under the best fit amino acid substitution model identified as the WAG+Γ+I for VP1 and VP2, and JTT+Γ for putative VP3 protein data set using jModelTest 2 [32]. Monte Carlo Markov chains (MCMC) were run for 107 generations, sampling every 1000 trees. Traces were inspected for convergence with Tracer 1.5 (https://github.com/beast-dev/tracer/releases). Maximum clade credibility (MCC) trees for each ORF were generated and visualized using FigTree v1.4.1 (http://tree.bio.ed.ac.uk/software/figtree/).

## 3. Results

### 3.1. Molecular Screening of GyV11

An incomplete genome of a novel gyrovirus, named GyV11 (1855 b fragment) was assembled from one of the five cloacal pools subjected to deep sequencing. In that pool, only one individual, a ferruginous-backed antbird (*Myrmeciza ferruginea*), tested positive, so this individual’s cloacal sample was subjected to a second metagenomic analysis in order to fully sequence the complete genome. The same ferrugineous-backed antbird also tested positive for GyV11 in the blood sample, but not in the oral sample. In the 356 additional cloacal samples that were not subjected to deep sequencing, the identical GyV11 sequence was also found in the oral sample from a rufous-rumped foliage-gleaner (*Philydor erythrocercum*) and in the cloacal sample of a white-plumed antbird (*Pithys albifrons*). All of the positive results were confirmed by Sanger sequencing. Hence, 3 out of 406 birds (0.73%) were positive for GyV11, all three of them sampled during the dry season. Attempts to isolate GyV11 were not possible owing to very limited amount of each sample available.

### 3.2. Genomic Characterization

**Figure 1:**
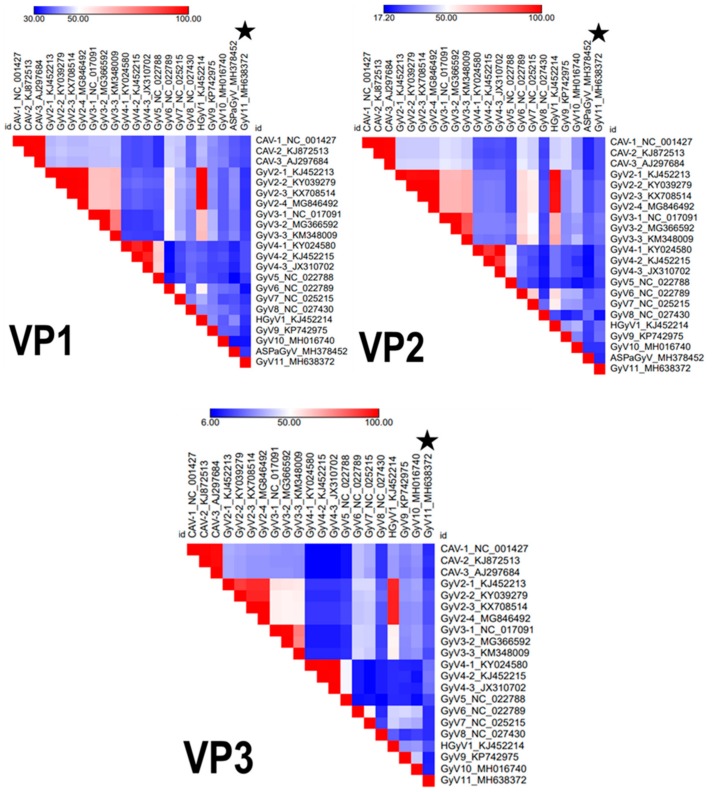
Heatmaps created from pairwise similarities for the three viral proteins (VP1, VP2, and VP3) among all representative gyroviruses. Legend values show in percentage the lowest (dark blue) and the highest (red) similarities in the comparison. CAV: chicken anemia virus; GyV: gyrovirus; HGyV: human gyrovirus.

The genomic sequence of this virus differs considerably from all other gyroviruses previously described (shared amino acid similarity of 25% to 29% for VP1, 13% to 21% for VP2, and 10% to 17% for VP3; Figure 1).

**Figure 2:**
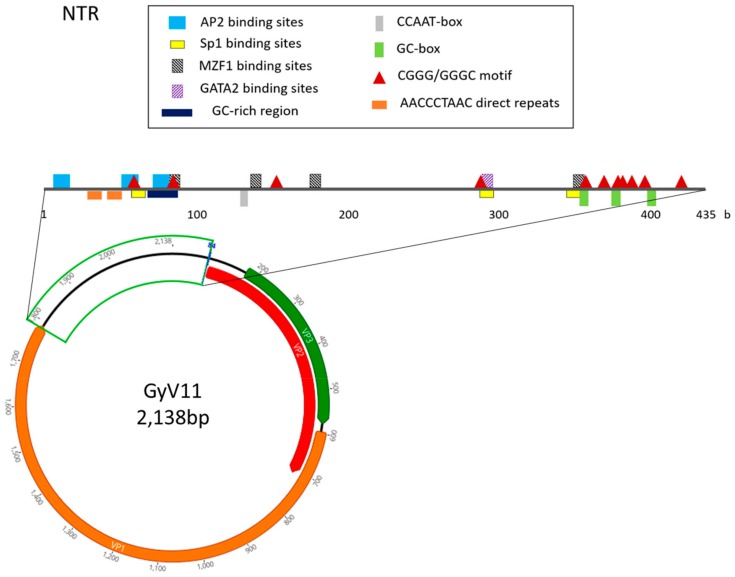
Genomic organization of the gyrovirus GyV11 with the three characteristic open reading frames of the members of the genus Gyrovirus: VP1 (capsid protein), VP2 (phosphatase), and VP3. The non-translated region (NTR) is highlighted and the positions of the putative regulatory motifs are shown. MZF, myeloid zinc finger; Sp1, zinc finger transcription factor.

The genome of GyV11 is a circular DNA molecule, 2138 bases long, which exhibits the three characteristic ORFs of gyroviruses, VP1, VP2, and a putative VP3 partially overlapping each other (Figure 2). VP1, VP2, and VP3 amino acid sequence comparisons revealed that, while VP1 and VP2 are somewhat similar to those previously described, VP3 shows little similarity to other gyrovirus VP3. GyV11 VP1 encodes a putative viral capsid protein with 397 aa. This VP1 shows a typical N-terminal region rich in basic amino acids with high arginine content typical of capsid proteins. The VP2 protein is 206 aa long and contains the WX_7_HX_3_CXCX_5_H and CX_5_R motifs (at positions 30 and 42, respectively) characteristic of protein tyrosine phosphatases in gyroviruses and other anelloviruses. The VP3 ORF encodes a protein of 134 aa, being the longest VP3 among gyroviruses described so far. The putative VP3 in GyV11 did not contain apoptin conserved protein domains. However, we found three motifs that are common in other gyrovirus VP3: a proline-rich region in the N-terminal region, a bipartite nuclear localization sequence (RKRPKPGTEAWLLQRKKE; position 90), and a putative nuclear export sequence (LECNEIL; position 117), with the latter two located in the C-terminal region.

The non-translated region (NTR) of the GyV11 is 435 bp in length (Figure 2) and shared an unusual constellation. Up to 11 putative transcription factor binding sites were identified: four for MZF1 (myeloid zinc finger 1) (positions 82, 136, 174, and 348), three for Sp1 (zinc finger transcription factor) (positions 58, 286, and 345), three for AP2 (positions 6, 51, and 71), and one for GATA2 (position 287). However, no canonical TATA boxes were identified. Two 4-b motifs (CGGG and GGGC) previously described in torque teno viruses were found 11 times along the NTR (positions 55, 81, 148, 283, 353, 364, 373, 376, 383, 391, and 415). Three of these motifs are located in the three GC boxes found (positions 352, 373, and 397) (Figure 2). Four putative stem-loop structures were observed between positions 250 and 370. Furthermore, a polyadenylation signal (AATAAA; position 24) and two direct repeats (5’-AACCCTAAC-3´) connected by 4 b are located at the 5´ region of the NTR. A GC-rich region is also located in this 5´ region of the NTR, between positions 68 and 88. The promoter nucleotide motif containing a putative estrogen-response element (ACGTCA) detected in several gyroviruses [14] that can upregulate transcription was found in the GyV11 NTR. The findings suggest that this virus represents a novel member in the genus Gyrovirus, and thus we propose it as a new gyrovirus species or genotype, designated as GyV11 (GenBank accession number MH638372).

### 3.3. Phylogenetic Analysis

**Figure 3:**
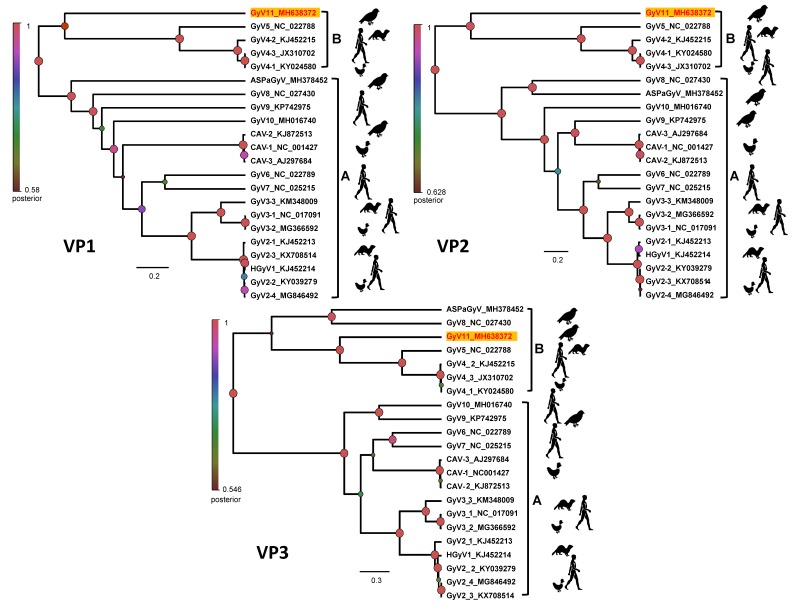
Bayesian maximum clade credibility (MCC) trees representing the phylogenetic reconstructions of the gyroviruses including the GyV11 from this study based on the amino acid sequences of VP1, VP2, and VP3 proteins. Bayesian posterior probabilities are indicated at the nodes. Strain names and GenBank accession numbers for sequences used to construct the trees are indicated on the branches. The scale bar indicates mean number of amino acid substitutions per site.

The Bayesian maximum clade credibility (MCC) trees and maximum likelihood phylogeny (not shown) of gyrovirus VP1, VP2, and putative VP3 amino acid sequences revealed two well supported lineages of gyroviruses (Figure 3), corresponding to gyrovirus groups A and B [14]. The molecular phylogenies showed that GyV11 belongs to the gyrovirus lineage B, with VP1 and VP2 sequences being markedly divergent and forming a basal node with respect to previously reported gyroviruses of that lineage, GyV4 and GyV5, detected in humans, ferrets, and fowl. The VP3 sequence is also most closely related to GyV4 and GyV5, with which it forms a sublclade that is sister to two gyroviruses found in sea birds (GyV8 and ASPaGyV). The most recent common ancestor of the two clades is supported by posterior and bootstrap values (Figure 3). The position of GyV11 in all trees showed a sister relationship and is consistent with the genetic equidistance to all viruses from the lineage B (Figure 1 and Figure 3). In addition, the phylogenetic analyses also revealed a basal position of all gyroviruses detected in wild birds, likely owing to ancestral origin and longtime evolution.

## 4. Discussion

In this study, we have detected and characterized for the first time the presence of a novel gyrovirus, GyV11, in wild populations of birds in a remote area with practically no human influence. Owing to very low similarities with previously known gyroviruses, GyV11 could be designated as a novel gyrovirus species. Moreover, it is also the first time that the same gyrovirus is found in individuals from three different avian species. To date, the study of gyroviruses in birds has been almost exclusively limited to poultry [11,13,33], with GyV8, GyV10, and ASPaGyV being the only ones described exclusively in other avian species (*Fulmarus glacialis*, *Chauna torquata*, and *Oceanodroma homochroa*, respectively) [17,18]. Both GyV8 and GyV10 were identified in diseased birds, although GyV10 was also present in apparently healthy individuals [18]. GyV8 was found in one northern fulmar showing ataxia and head tilt, but no other fulmar with similar clinical signs analyzed resulted positive for GyV8 [17]. ASPaGyV was discovered in cloacal samples from ashy storm petrels [19]. The presence of GyV11 was detected in three different birds from the Nouragues Natural Reserve, but only one of them was found to be positive in both cloacal and blood samples, thus supporting the idea of a possible systemic infection. The concurrent presence of CAV in blood and rectal content has been previously shown to occur in chicks inoculated with the virus at one day of age up to seven days after inoculation [6]. The bird likely infected by GyV11 was also found to be positive for astrovirus in the cloacal sample, which may suggest a compromised immune system and a possible co-infection. Co-infections with Marek’s disease virus and CAV and with GyV2 and Newcastle Disease Virus have been reported in chickens [34,35]. The other two birds from Nouragues resulted positive for GyV11 only in oral or cloacal samples, but not in blood, so the existence of infection cannot be confirmed. However, the presence of this new gyrovirus in oral and cloacal samples from different bird species strengthens the hypothesis that the fecal-oral route is likely the main route of transmission for gyroviruses [6]. Similarly to mammals, the impact of gyroviruses in the bird’s health is questionable as they can represent active infections or passive dietary transit [15]. However, our study shows that GyV11 can be dispersed by the faeces of, at least, two different avian species (*Myrmeciza ferruginea* and *Pithys albifrons*) and is likely circulating among different bird species within the same community. In fact, in a closer area such as Brazil, the circulation of a phylogenetically close genotype (GyV4) has been detected in chickens and three other gyroviruses (CAV, GyV2, and GyV3) have been found in the fecal virome of wild birds [3,36].

The prevalence of GyV11 in the analyzed Neotropical birds (0.73%) is much lower than the prevalence of other gyroviruses observed in poultry [11,37,38]. A serological survey implemented in Japan showed that anti-CAV antibodies were present in chickens (60.2% seroprevalence) and quails (61.3%), but they were not found in blood samples from wild birds [39]. To our knowledge, there are no other prevalence studies of gyroviruses in wild bird populations. However, the proportion of positive birds in our study is similar to that of other avian viruses analyzed in the same Neotropical rainforest [40]. This could be explained because the conditions in broiler farms—enclosures with a high density of individuals of the same species—likely favor a faster spread of any infection by avian pathogens than in the pristine rainforest studied here. In addition, it has been shown that CAV and GyV2 are present as contaminants in some commercial poultry vaccines [41], which could explain seroprevalence values as high as 100% in some chicken flocks for CAV [37] and the widespread distribution of GyV2 [11].

Gyroviruses have also been detected in faecal samples from other vertebrates such as ferrets, cats, and humans [12,13,14,16]. Prevalence studies carried out in human stools showed values more similar to those observed in ours (0.56 and 1.67% in children with diarrhea) [14], although some others show higher prevalences (13.9–18.9% in patients with diarrhea) [13]. However, the majority of these viruses showed high similarity to gyroviruses previously described in chickens, so the presence of these viruses in the feces is likely owing to consumption of infected chicken meat. This hypothesis is reinforced by two studies carried out to find gyroviruses in human blood in Italy and France [8,9]. Recent studies found that HGyV1 was mainly present in immunocompromised patients and, though it was detectable among healthy individuals, its prevalence was low (0.85%). However, the discovery of new gyrovirus genomes may improve the molecular detection of different gyrovirus types in human samples and those prevalences could turn out to be higher.

Regarding the GyV11 genome, we found a high number of putative regulatory motifs, but only one of them (Sp1 binding sites) had been previously described in the NTR of other gyroviruses [42]. The number of direct repeats in the NTR is also different from that of, for example, CAV, where in most isolates four direct repeats with a 12 b insert can be found [42]. However, four putative stem-loop structures that could act as transcription enhancers were found in the NTR of GyV11. It is noteworthy that two 4-b motifs (CGGG and GGGC), common in the NTR of other anelloviruses, the torque teno viruses, have been found 11 times along this region, some of them adjacent to the stem-loop motifs, as it happens in torque teno viruses [43]. The presence of five putative binding motifs for two transcription factors related to hematopoiesis such as MZF1 and GATA2 is also remarkable [44,45]. Whether these differences cause an increase in viral fitness in regards to establishment and spatial diffusion requires further analysis.

GyV11 VP1 and VP2 are similar to those of other identified gyroviruses. Genetic analysis revealed that VP1 has a N-terminal region rich in basic amino acids, a characteristic shared by all members of the Anelloviridae family [5], and that VP2 has the CX_5_R and the WX_7_HX_3_CXCX_5_H motifs characteristic of protein tyrosine phosphatases, conserved in other gyroviruses and anelloviruses [5,46]. However, VP3 nucleotide and amino acid sequences showed little similarity to those of previously described gyroviruses. The protein encoded is comprised of 134 aa, and is the longest VP3 among gyroviruses described to date. This ORF overlapping VP2 has been identified in all gyroviruses except GyV4 [17]. In spite of this, the protein it encodes has some important motifs related to the apoptotic function of VP3 in CAV: a proline-rich region in the N-terminal, a nuclear localization sequence, and a putative nuclear export sequence [47,48]. Taking this into account, we cannot rule out the hypothesis that the VP3 of GyV11 may also induce apoptosis or, at least, play a role in the cell nucleus, regardless of the dissimilarity its sequence has with the other VP3 described.

Phylogenetic and genetic distance indicated that GyV11 represent a new prototype for another gyrovirus species. This study further supports the existence of two distinct gyrovirus lineages, A and B. As previously described [14], we also found several genomic characteristics supporting the presence of two distinct gyrovirus groups. The group A VP1s were substantially longer than those of group B. Furthermore, all members of lineage B genomes contained a small putative VP3 in the same location as the VP3/Apoptin protein present in genome of all members of lineage A, but in different reading frames. Evolutionary relationships seem particularly uncertain when it comes to those gyroviruses found in other avian species apart from chickens, that is, GyV8, GyV9, GyV10, and GyV11. Thus, the discovery of new members of the Gyrovirus genus together with a phylogenetic consensus are needed to clarify evolutionary processes in this group of viruses. Phylogenetic analysis also indicates that most basal gyroviruses in both A and B lineages are found in wild bird species, suggesting that gyroviruses may have originated in wild bird populations before spreading to other organisms.

In conclusion, GyV11 represents a novel member of the genus *Gyrovirus*, which shows little similarity with other previously described members of the genus. It was detected in cloacal, oral, and blood samples from passerines in a remote rainforest in French Guiana. The presence of the GyV11 in faeces and blood could be considered as responsible for a possible immunosuppression status, as has been previously shown for other gyroviruses such as CAV and GyV10. The detection and genetic characterization of a novel gyrovirus (GyV11) from a remote area expands our knowledge about the geographic distribution, host range, ecology, diversity, genomic structure, and evolutionary relationships of gyroviruses.

## Supplementary Materials

The following are available online at https://www.mdpi.com/1999-4915/11/12/1148/s1, Table S1: List of species including the 50 individuals selected for deep sequencing and the 356 additional individuals tested for prevalence analysis.
