# Supplementary material for: A Novel and Divergent Gyrovirus with Unusual Genomic Features Detected in Wild Passerine Birds from a Remote Rainforest in French Guiana
Source: Viruses. 2019 Dec 11;11(12):1148. doi: 10.3390/v11121148 (PMC6950609; doi:10.3390/v11121148)
Supplement: Supplementary file 1 [file viruses-11-01148-s001.pdf]

Table S1. List of species including the 50 individuals selected for deep sequencing and the 356 additional individuals tested for prevalence analysis.

Deep sequencing

**Species**	**Number of individuals**
Glyphorynchus spirurus	8
Gymnopithys rufigularis	2
Hylophylax naevius	2
Lepidotrix serena	2
Leptotila rufaxilla	1
Mionectes macconnelli	2
Philydor erythrocercum	3
Bucco capensis	1
Chloroceryle inda	1
Corapipo gutturalis	1
Corythopis torquatus	1
Dixiphia pipra	2
Myrmotherula longipennis	1
Pithys albifrons	2
Ramphocelus carbo	2
Cyanocompsa cyanoides	1
Formicarius analis	1
Hylophylax poecilinotus	1
Micrastur ruficollis ssp. *concentricus*	1
Myrmeciza ferruginea	2
Myrmotherula guttata	1
Pipra erythrocephala	1
Platyrinchus coronatus	1
Platyrinchus saturatus	1
Thamnomanes ardesiacus	2
Tachyphonus surinamus	1
Terenotriccus erythrurus	1
Thamnomanes caesius	1
Myrmotherula gutturalis	1
Turdus albicollis	1
Xenops minutus	1
Xiphorhynchus pardalotus	1

Prevalence analysis

Species	Number of individuals
Attila spadiceus	1
Automolus ochrolaemus	3
Celeus elegans	1
Cercomacra tyrannina	1
Chloroceryle inda	2
Conopophaga aurita	2
Corapipo gutturalis	4
Corythopis torquatus	3
Cyanerpes caeruleus	1
Cyanocompsa cyanoides	1
Cyphorhinus arada	1
Deconychura longicauda	1
Dixiphia pipra	25
Formicarius analis	1
Formicarius colma	2
Galbula albirostris	1
Glyphorynchus spirurus	30
Gymnopithys rufigularis	12
Hylophilus ochraceiceps	3
Hylophylax naevius	5
Hylophylax poecilinotus	22
Hypocnemis cantator	2
Lepidotrix serena	10
Leptotila rufaxilla	2
Lipaugus vociferans	2
Manacus manacus	5
Microbates collaris	2
Microcerculus bambla	1
Microrhopias quixensis	1
Mionectes macconnelli	18
Myiobius barbatus	1
Myrmeciza ferruginea	1
Myrmornis torquata	3
Myrmotherula axillaris	8
Myrmotherula guttata	5
Myrmotherula gutturalis	6
Myrmotherula longipennis	12
Myrmotherula menetriesii	2
Onychorhynchus coronatus	1
Oryzoborus angolensis	1
Percnostola rufifrons	4
Philydor erythrocercum	2
Philydor pyrrohodes	2
Phoenicircus carnifex	1
Pipra erythrocephala	6

Pithys albifrons	53
Platyrinchus coronatus	4
Platyrinchus saturatus	1
Progne chalybea	1
Ramphocaenus melanurus	2
Ramphocelus carbo	12
Rhynchocyclus olivaceus	1
Saltator maximus	1
Schiffornis turdina	3
Sclerurus caudacutus	3
Sclerurus rufigularis	9
Tachyphonus surinamus	4
Thamnomanes caesius	16
Thamnomanes ardesiacus	1
Thamnophilus murinus	2
Thryothorus coraya	2
Tolmomyias poliocephalus	1
Turdus albicollis	7
Tyrannus melancholicus	1
Xenops minutus	3
Xiphorhynchus pardalotus	3
